# Local Flap Reconstructions in Oral Cavity Defects: An Insight from 104 Cases

**DOI:** 10.5041/RMMJ.10526

**Published:** 2024-07-30

**Authors:** Poonam Joshi, Manasi Bavaskar, Rathan Shetty, Arjun Singh, Sudhir Nair, Pankaj Chaturvedi

**Affiliations:** Department of Head and Neck Surgical Oncology, Tata Memorial Centre, ACTREC, HOMI Bhabha National Institute (HBNI), Mumbai, India

**Keywords:** Local flaps, oral cancer defect, reconstruction

## Abstract

**Background:**

Resection of oral cavity carcinoma often leads to complex defects causing functional and aesthetic morbidity. Providing optimum reconstruction with free flaps becomes challenging in a high-volume center setting with constrained resources. Hence, understanding the local flap technique for reconstructing oral cancer defects is prudent.

**Materials and Methods:**

This study is a retrospective analysis of prospectively operated cases of oral cavity resections which were subsequently reconstructed using local flaps from 2019 to 2022. Patients who underwent reconstruction with either melolabial flap, islanded facial artery myomucosal (FAMM) flap, submental flap, supraclavicular artery island flap, infrahyoid flap, or platysma myocutaneous flap (PMF) were included in this analysis. Eligible patients were followed up to evaluate functional outcomes like oral feeding and to analyze the Performance Status Scale for Head and Neck Cancer.

**Results:**

The study included 104 patients. The tongue was the most common subsite, resulting in most hemiglossectomy defects, which were reconstructed using the melolabial flap procedure. Buccal mucosa defects in our series were reconstructed using the supraclavicular flap, whereas the submental flap procedure was the choice for lower lip-commissure defects. Complications such as partial and total flap loss, deep neck infection, and donor site complications like infection and gaping, oral cutaneous fistula, parotid fistula, and seroma were analyzed; the supraclavicular flap presented with a majority of complications.

**Conclusion:**

Local flaps are an alternative to free flap reconstruction in select cases with optimum functional outcomes and minimal donor site morbidity. This article comprehensively reviews the surgical steps for various local flap procedures in oral cancer defects.

## INTRODUCTION

Oral carcinoma is the most common cancer among males in India.^1^ Treatment involves resections that lead to complex defects, causing significant changes in functional and aesthetic outcomes and affecting the quality of life of patients.^2^ Planning a flap procedure includes precise analysis of the defect and the quality and quantity of tissue available for reconstruction. Minor defects can undergo primary closure, whereas medium-sized and large defects require local, regional, or free flaps for reconstruction.^3^–^5^ The choice of reconstruction differs for each subsite. One-third of defects following tongue carcinoma resection can be primarily closed due to the availability of sufficient mobile tissue. However, this may not apply to small buccal mucosa defects, where primary closure could lead to trismus. Larger defects may require local or free flap reconstruction to provide bulk and contour.

Local flaps are harvested near the defect and usually provide color and texture matching. They are relatively easy and quick to harvest, suitable for patients with significant comorbidities, and do not need special instruments or skills.^6^–^8^ In selected cases, studies have shown that local flaps have similar functional outcomes and complication rates as compared to free flaps.^3^,^4^ Nevertheless, replacing “like with like” remains the ultimate goal in achieving optimal oral defect reconstructions. Functional outcomes with free flaps are superior in cases where large, complex, and osseous reconstruction is required. Nevertheless, local flaps are a good option in selected medium-sized defects with minimal donor site morbidity.

In settings where a significant discrepancy exists between the high volume of patients and the number of free flap reconstructions, there is an unmet need for appropriate flap reconstructions. From this perspective, the utilization of local flaps in various sub-sites of the oral cavity, without comparisons to free flap reconstructions, merits exploration. Hence, this study retrospectively reviews the various types of local flaps utilized at our Institute and provides a comprehensive overview of the related surgical techniques, indications, and complications.

## MATERIAL AND METHODS

This study was a retrospective analysis utilizing prospectively collected data from all patients who underwent oral cavity resections and local flap reconstructions from 2019 to 2022. Patients with squamous cell carcinoma of the buccal mucosa, tongue, mouth floor, and lip were included in this study. Patients with large, complex defects involving skin and requiring segmental osseous resections were excluded from the study. All patients were assessed using functional outcomes such as oral feeding and duration of tracheostomy (postoperative day of tracheostomy tube corking), and the Performance Status Scale for Head and Neck Cancer (PSS-HN).

The need for informed patient consent was waived due to the retrospective nature of the study. The study was conducted according to the ethical guidelines outlined in the Declaration of Helsinki,^9^ Good Clinical Practice guidelines,^10^ and the Indian Council of Medical Research^11^ guidelines. Institutional ethics committee approval was obtained (IEC3 900849).

The assessed complications included partial flap loss, total flap loss, deep neck infection, donor site complications (e.g. infection and gaping), and miscellaneous complications (e.g. oral cutaneous fistula, parotid fistula, and seroma). Medical data were retrieved from the Institute’s electronic medical record system, and patient demographics as well as clinical, radiological, pathological, and treatment details were recorded. The collected data were analyzed using SPSS 22 software.

## RESULTS

This study included 104 patients with a male-to-female ratio of 6:1 (90:14). The mean age of the population was 52 years (range 28–75 years). All patients were Eastern Cooperative Oncology Group (ECOG) Performance Status 0 or 1. The majority of patients presented with no comorbidities or general systemic conditions; a subset of patients had comorbidities such as hypertension (*n*=6; 5.8%) and diabetes (*n*=4; 3.9%). Table 1 elaborates on the demographic and disease-related data of the study cohort. Of note, the majority of patients had early-stage squamous cell carcinoma (pT1–T2); 78 (75%) were node-negative, and 62 (59.6%) required adjuvant treatment. All the pT4 cases had tongue carcinomas. Nine underwent melolabial flap reconstruction, and one underwent infrahyoid flap reconstruction.

**Table 1:** Demographic and Clinicopathologic Details.

Patient Features	Value
Total patients, *n*	104

Age	
Median, y	53
Range, y	28–75

Gender	
Male, n	90
Female, n	14

Disease site	
Tongue, n (%)	57 (54.8%)
Buccal mucosa, n (%)	36 (34.6%)
Lip, n (%)	9 (8.7%)
FOM, n (%)	2 (1.9%)

Pathological tumor stage	
pT1, n (%)	15 (14.5%)
pT2, n (%)	51 (49.0%)
pT3, n (%)	28 (26.9%)
pT4, n (%)	10 (9.6%)

Pathological nodal stage	
pN0, n (%)	78 (75.0%)
pN1, n (%)	7 (6.7%)
pN2, n (%)	14 (13.5%)
pN3, n (%)	5 (4.8%)

Adjuvant treatment	
None, n (%)	42 (40.4%)
Adjuvant radiotherapy, n (%)	45 (43.3%)
Adjuvant chemoradiotherapy, n (%)	17 (16.3%)

FOM, floor of mouth; pN, pathological nodal; pT, pathological tumor.

Out of the 57 patients (54.8%) with tongue carcinoma, hemiglossectomy was performed in 28 (49.1%) patients, anterior two-thirds glossectomy in 18 (31.6%), and one-third glossectomy in 11 patients (19.3%). Likewise, 36 patients (34.6%) had buccal mucosa cancer, which resulted in 12 patients (33.3%) undergoing buccal mucosa-wide excision, and 24 patients (66.7%) requiring marginal mandibulectomy. The flap types used in all patients are listed in Table 2. The melolabial flap was the most commonly used flap.

**Table 2:** Flaps Used for Surgical Interventions in 104 Patients.

Flap	Number of Patients (%)
Melolabial flap	63 (60.6%)
Supraclavicular flap	16 (15.4%)
Submental flap	11 (10.6%)
Islanded FAMM flap	5 (4.8%)
Infrahyoid flap	5 (4.8%)
Platysma flap	4 (3.8%)

The time needed to harvest all flaps ranged from 45 to 55 minutes. However, the time to perform an islanded facial artery myomucosal (FAMM) flap procedure ranged from 55 to 70 minutes, indicating that this procedure requires additional time. This can be attributed to the small harvest area along the course of the facial artery and vein.

The complications associated with all the flaps are summarized in Table 3. Partial necrosis (<50% flap loss) was the most common complication encountered in 8 patients (7.7%). All patients were managed conservatively with debridement and healing via secondary intention. Total flap necrosis was seen in 2 patients (1.9%), one with the supraclavicular flap and the other with the islanded FAMM flap. These patients were managed using skin grafting and primary closure, respectively. All the patients with flap necrosis were successfully healed with conservative management without a need to redo the flap. Donor site complications such as wound gaping, oral cutaneous fistula, and seroma occurred in six patients, all of whom were managed conservatively by suturing and dressings. The majority of complications were found in the supraclavicular flap.

**Table 3:** Complications and Management of All Local Flaps.

Complications (*n*)	Flap (*n*)	Management
Partial necrosis (8)	Supraclavicular (5) Infrahyoid (2) Platysma (1)	Debridement and secondary healing
Total necrosis (2)	FAMM (1) Supraclavicular (1)	Primary closure Skin grafting
Deep neck infection (5)	Melolabial (1) FAMM (1) Supraclavicular (2) Infrahyoid (1)	Antibiotic coverage and antiseptic washes
Donor site complications (wound gaping, parotid fistulas, seroma) (6)	Melolabial (1) FAMM (1) Supraclavicular (4)	Conservative management

FAMM, facial artery myomucosal.

The nasogastric tube remained in place for a mean duration of a mean duration of 12 days (range, 8–18 days). Only 14 patients (13.5%) had a tracheostomy; tube corking was done on postoperative day 5 for all the patients.

Based on the PSS-HN measures at 6 months follow-up, 73 patients (70.2%) were on a complete diet (liquid assist), and 31 patients (29.8%) were on a full diet (no restriction). Furthermore, 86 patients (82.7%) achieved normal oral intake in public. Understandable speech was achieved in 84 patients (80.8%), with only occasional repetition being necessary.

## DISCUSSION

There are several classification systems for oral defects. While Schultz et al.^12^ and Boyd et al.^13^ established a classification system for mandibular defects, Bhattacharya et al.^14^ classified tongue defects. However, as most defects include composite defects of the tongue, mandible, and buccal mucosa, the classification system by Liu et al.^15^ fulfills all requirements. Nonetheless, its complexity can make it challenging in day-to-day practice. For the present study, the classification system of Squaquara et al.^16^ was used to define defect sizes. Defects were classified as small (up to 4 cm maximum diameter), medium (up to 7 cm), and large (more than 7 cm).

Other than defect size, the subsite involvement, type of tissues involved (mucosa/skin/bone), functional dynamics (e.g. resection of a mobile structure like the tongue), and prosthetic rehabilitation following bone resection must be considered. Free flaps are the gold standard for reconstructing medium and large defects. However, medical comorbidities, the surgeon’s training, patient preferences, donor site morbidity, and availability of resources for free flaps are major limiting factors. Hence, the reconstructive elevator concept can be adopted. The choice of a reconstruction methodology should be based on which one is simplest and provides the best functional outcomes.

Local flaps are particularly useful for small to medium-sized oral defects. The melolabial flap is robust, having adequate soft tissue bulk for volume restoration of the oral cavity and, occasionally, for oropharyngeal reconstruction.^17^ The resulting scar is also well masked in the nasolabial crease. The pliability and versatility of the FAMM flap make it an excellent choice for reconstruction of the tongue, floor of the mouth, and palatal defects. This flap is reliable, has a strong vasculature, and provides mucosal replacement for mucosal tissue defects with no concern for hair growth.^18^

The submental flap is a promising reconstructive option in clinical-radiologically determined N0 of the neck. It is easy to harvest with no need for a second-stage flap division.^19^ The supraclavicular artery island flap is thin, pliable, with good length, and an excellent color match for complex oral cancer defects.^20^ The infrahyoid flap is reliable for lower oral cavity reconstructions such as floor of mouth defects, and has low surgical complexity.^21^ Lastly, the platysma myocutaneous flap (PMF) has multiple advantages that have been under-recognized by surgeons for several years.^22^ It is thin, quick, and easy-to-harvest.

### Different Local Flaps for Oral Cavity Reconstruction

**Figure 1:**
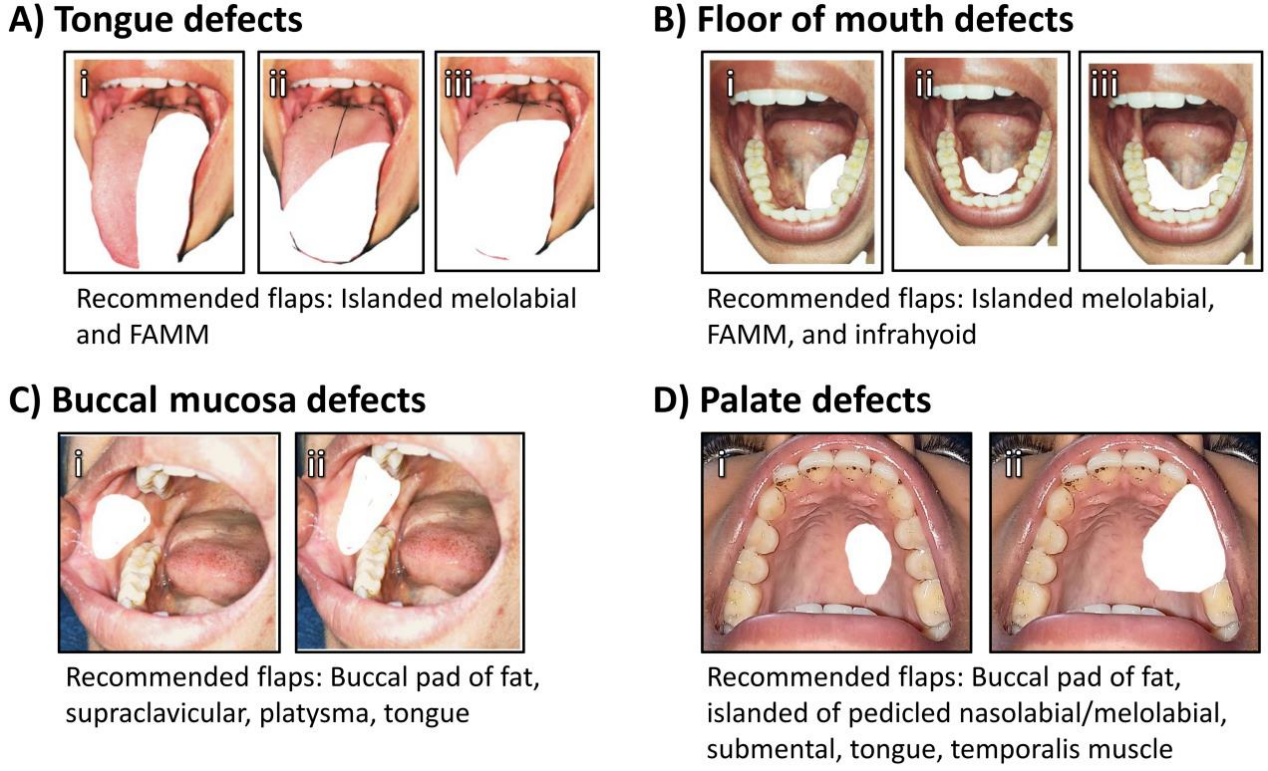
Types of Oral Cavity Defects after Resection and Recommended Local Flaps to Use for Reconstruction. For all panels, white indicates the defect. **A:** Tongue defects ahead of circumvallate papilla: (i) hemiglossectomy, (ii) extended hemiglossectomy, (iii) anterior two-thirds glossectomy. **B:** Floor of mouth defects: (i) small unilateral defect, (ii) small bilateral defect, (iii) medium-sized bilateral defect. **C:** Buccal mucosa defects: (i) Simple defect involving buccal mucosa, (ii) omplex defect involving buccal mucosa-gingivobuccal sulcus. **D:** Palatal defects: (i) small defect not involving the upper alveolus, (ii) defect involving resection of the upper alveolus.

Despite the pros and cons of each flap, careful case selection is essential when choosing a local flap as the reconstructive option. Different flaps present with different limitations such as a limited arc of rotation, less tissue availability, inadequate bulk, and nodal disease—which sometimes interferes with certain flaps. Figure 1 presents our recommended algorithm for determining which flap to use in reconstructions of the tongue, buccal mucosa, floor of the mouth, and palatal defects. The surgical technique for each flap, and our experience with its limitations, are described in detail below.

#### The melolabial flap

**Figure 2:**
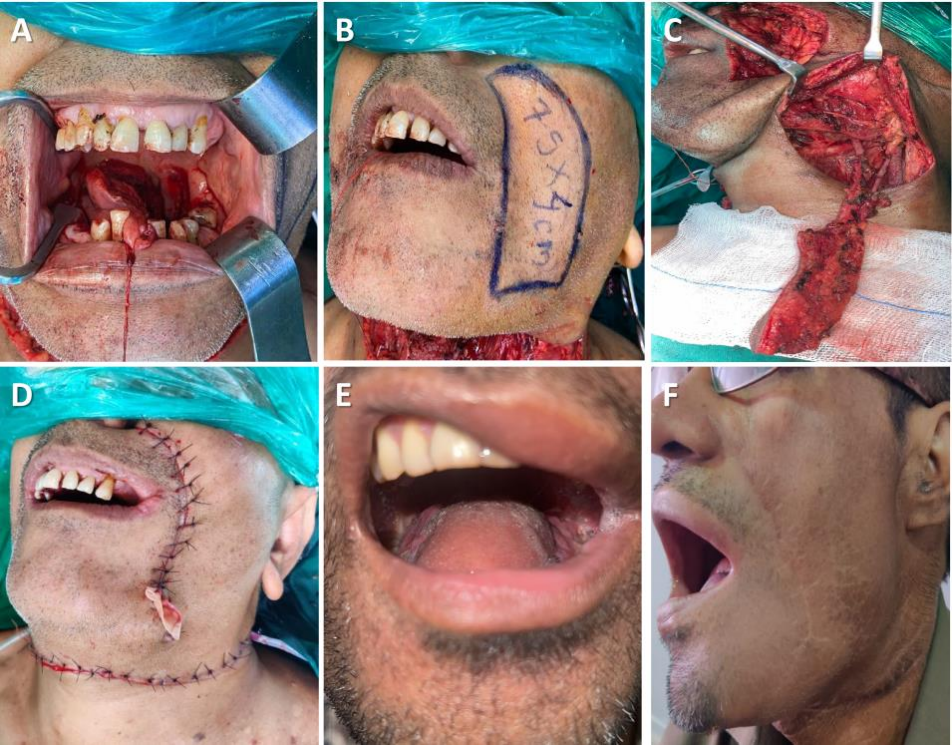
Melolabial Flap for Hemiglossectomy Defect Reconstruction. **A:** Defect size. **B:** Marking the flap. **C:** Melolabial flap islanded on facial vessels. **D:** Primary closure of donor site. **E:** Flap inset (outcome after 1 month). **F:** Donor site scar hidden in the nasolabial crease (after 3 months).

Figure 2 shows the steps for a melolabial flap reconstruction and postoperative pictures.

Facial artery- and vein-preserving neck dissection is done. After assessing the defect size and skin pliability, the flap is marked in the nasolabial crease. The aim of the reconstructive procedure is to achieve tension-free closure of the donor site while avoiding stretching of the lip angle. First, a lateral incision is made through the skin and subcutaneous tissue, and deeper to the zygomaticus major and minor muscles, since the facial vessels lie beneath the upper part of the facial mimetic muscles. The facial vein, which has a straighter course, is identified and clipped. Next, the medial incision is made, and the facial artery (anterior to facial vein) is identified lateral to the buccinator muscle. The labial, mental, and platysmal branches from the artery are identified and clipped. In addition, Stenson’s duct (and the buccal branch of the facial nerve) is identified and preserved. The flap is then elevated and transferred to the neck underneath the marginal mandibular nerve through a subcutaneous tunnel lateral to the mandible.

The islanded melolabial flap is quite versatile and can be used for tongue, floor of mouth, lip, alveolus, and palatal defect reconstructions. Its dimensions vary from 2.5 cm to 4 cm wide and 8 cm to 10 cm long. However, caution should be exercised when considering a flap >4 cm wide, which can result in stretching and deviation of the lip. The term “melolabial” is preferred over “nasolabial” since the melolabial region comprises tissue over the cheek area that extends from the nasal ala to the oral commissure, corresponding to the area from where the flap is raised.^23^

In our experience, the major challenge encountered was transfer of the flap into the neck underneath the marginal mandibular nerve. Transient nerve paresis was seen in 24 out of 63 patients (38.1%) immediately post-surgery. However, none of these patients developed permanent nerve palsy at 6 months follow-up. Another hurdle experienced was the constant caution during surgery to stay above the buccinator muscle and to identify the minor salivary glands; failure to do so leads to accidental entry into the oral cavity.

#### The islanded FAMM flap

**Figure 3:**
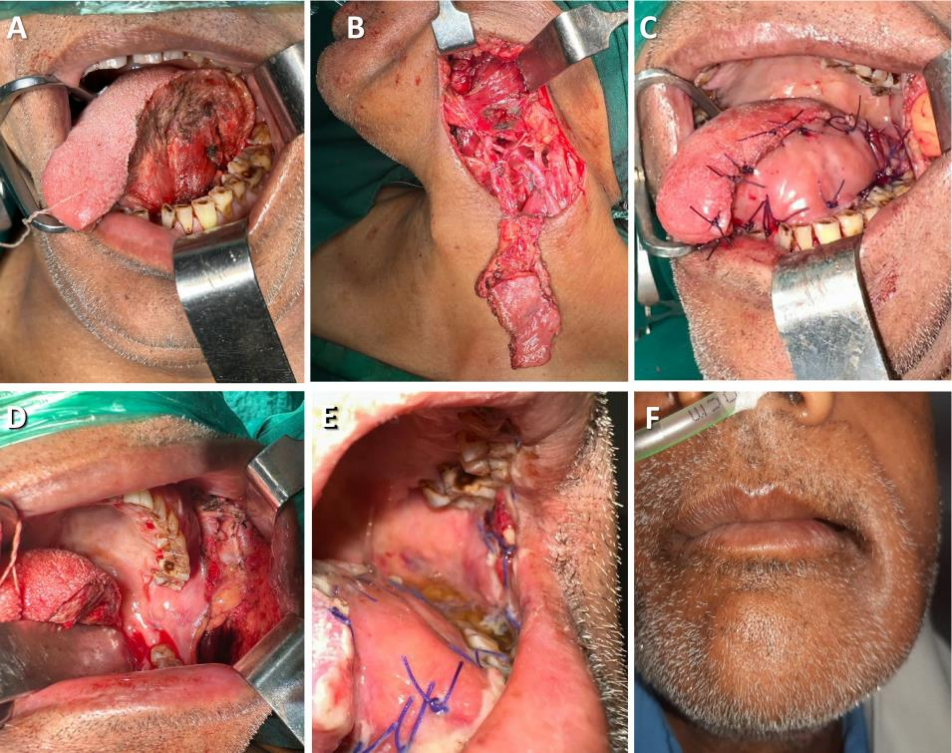
Islanded Facial Artery Myomucosal Flap (FAMM) for Hemiglossectomy Defect Reconstruction. **A:** Defect size. **B:** Flap islanded on facial vessels. **C:** Flap inset. **D:** Donor site closed with buccal fat pad. **E:** Recipient and donor site healing. **F:** Minimal skin puckering seen externally.

Figure 3 shows the steps for islanded FAMM flap reconstruction and postoperative pictures.

To access the FAMM flap, a facial artery- and vein-preserving neck dissection is done. The artery’s course is marked on the buccal mucosa using hand-held Doppler. Marking the flap extends from 1 cm behind the oral commissure to immediately anterior to the retromolar trigone. The flap width ranges from 2.5 to 3.5 cm. Using a sharp tip cautery, an anterior incision is made on the buccal mucosa at a plane deeper than the buccinator muscle. The facial artery is identified as lateral/deep to the buccinator muscle. The posterior incision is extended until it reaches beneath the buccinator muscle, and the flap is elevated in a plane deeper than the facial artery. The facial vein is identified in the lower part over the masseter muscle, which is then incorporated in the flap. The flap is dissected over the mandible and transferred to the neck beneath the marginal mandibular nerve. After tunneling through the lingual aspect of the mandible, the flap is repositioned into the defect. This technique is a single-stage procedure that provides excellent mobility to the flap.^24^ The donor site defect is covered with a buccal fat pad.

The islanded FAMM flap is an arterialized flap that relies on the facial artery for its blood supply. Incorporating the facial vein in the flap can lead to a better and more robust blood supply, with minimal chances of venous congestion and flap failure. We have experienced one complete flap loss when the vein was not included. All the flaps were fine after the inclusion of the facial vein.

#### The submental flap

**Figure 4:**
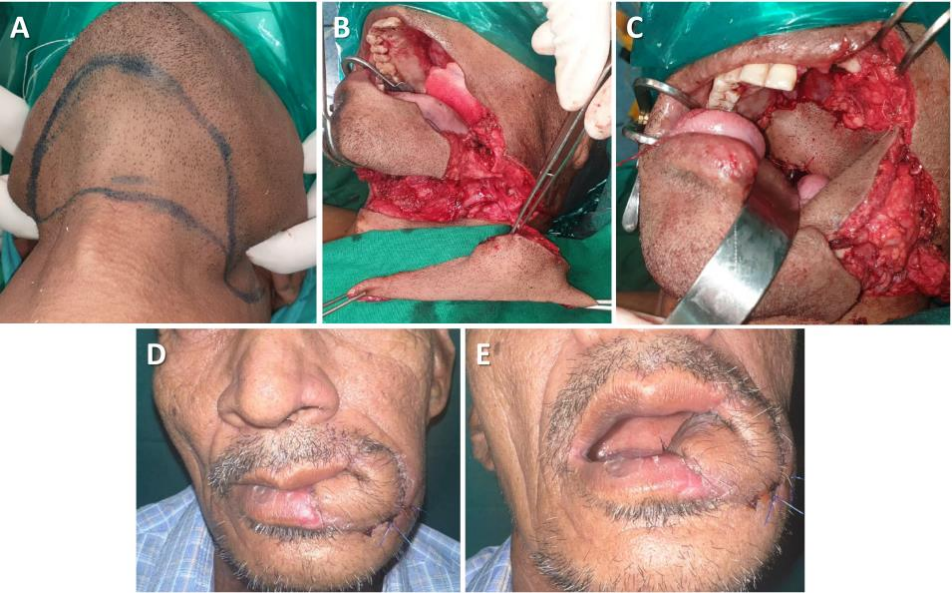
Submental Flap for Complex Buccal Mucosa Defect Reconstruction. **A:** Marking of flap. **B:** Flap islanded on submental vessels. **C:** Flap inset for complex buccal mucosa defect involving commissure. **D, E:** Outcome of flap (after 1 month).

Figure 4 shows the steps of submental flap reconstruction and postoperative pictures.

The submental flap is marked in the submental area and can be extended to both submandibular areas, from one angle of the mandible to another. The flap size varies from 6 cm × 5 cm to 11 cm × 6 cm. The skin is pinched to evaluate if the donor defect can be closed primarily after the flap is elevated. A neck dissection is performed preserving the facial and submental arteries and veins. The lower incision is taken through the skin, subcutaneous tissue, and platysma. The flap is raised in a subplatysmal plane. The facial vessels and submental artery in the submandibular area are carefully located by following their horizontal path between the mylohyoid muscle and the anterior belly of the digastric muscle. To preserve the thin terminal part of the sub-mental artery, part of the mylohyoid muscle, along with the anterior belly of the digastric muscle, is incorporated in the flap after detaching them from the mandible and hyoid. The upper incision of the flap is similarly taken below the platysma, and the flap is mobilized and completed. Finally, a subcutaneous tunnel is made lateral to the mandible and transferred to the oral cavity.

The submental flap is good for oral defect reconstructions. However, oncological nodal clearance of the neck is essential.^25^ The flap is easy to harvest, pliable, considerably large, and can be raised and reconstructed in a single-step procedure.^26^ Our group has considered submental flaps only in verrucous carcinoma with clinicopathological N0 necks.

#### The supraclavicular artery island flap

**Figure 5:**
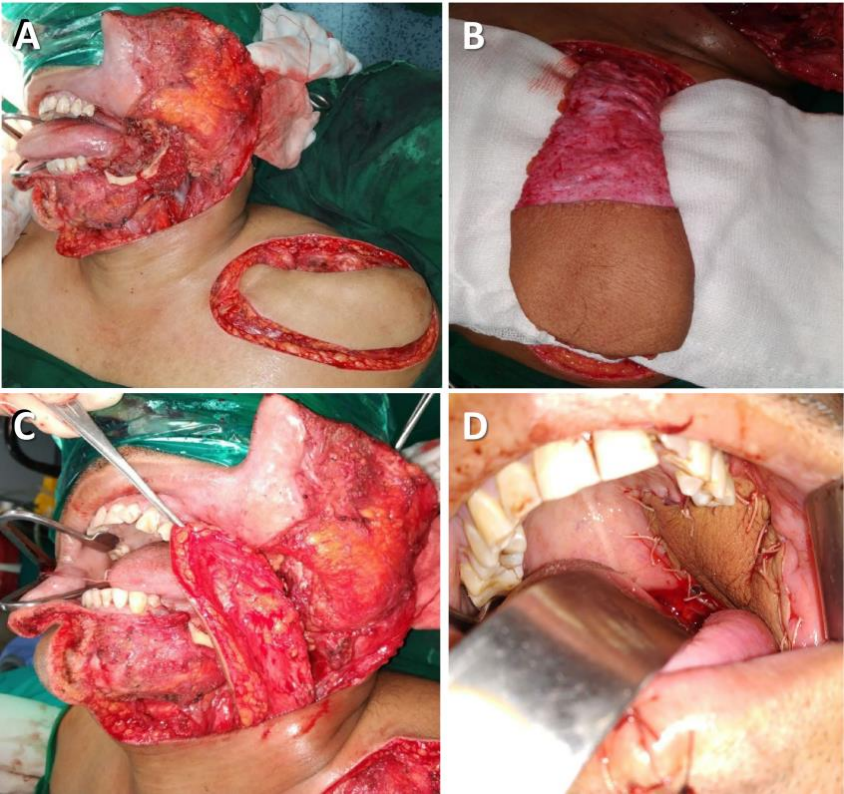
Supraclavicular Flap for Bite Marginal Defect Reconstruction. **A:** Bite marginal resection defect and marking of flap. **B:** Flap harvested. **C:** Flap tunneled into the defect. **D:** Flap inset.

Figure 5 shows the steps of the supraclavicular artery island flap reconstruction.

The supraclavicular artery island flap is marked in the supraclavicular area, with the artery marked in the triangle formed medially by the posterior border of the sternocleidomastoid muscle, laterally by the external jugular vein, and inferiorly by the clavicle. A 6 cm × 22 cm flap is marked after testing skin pinchability for primary closure. Marking of the lateral flap edge can be extended 2 cm lateral to the deltopectoral groove; beyond this care should be exercised as the blood supply to the flap becomes unstable. The flap is raised in a sub-fascial plane over the deltoid muscle and extended to the pivot point at the previously marked triangle. Care should be taken when performing a neck dissection at level IV to preserve the supraclavicular pedicle.

The supraclavicular artery island flap is fasciocutaneous. Color matching is excellent for neck and face defects, and donor site morbidity is minimal.^20^ However, using the distal part of this flap is usually precarious as it has an unreliable blood supply. In our series, five patients developed partial flap necrosis, and one developed total flap necrosis. All these flaps were debrided and managed conservatively. The patient with complete flap necrosis was managed with a skin graft.

#### The infrahyoid flap

**Figure 6:**
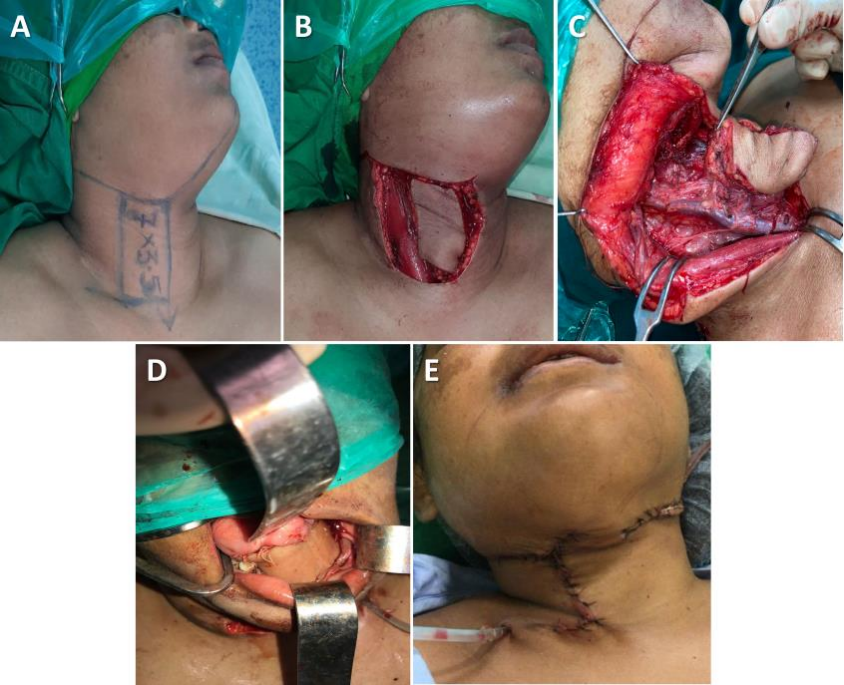
Infrahyoid Flap for Floor of Mouth Defect Reconstruction. **A:** Marking of flap. **B:** Flap elevation. **C:** Neck dissection completed and flap islanded on superior thyroid vessels. **D:** Flap inset into defect. **E:** Donor site closure.

Figure 6 shows the steps involved for using the infrahyoid flap.

The infrahyoid flap is based on the infrahyoid artery and vein arising from the superior thyroid pedicle.^27^ The flap is marked in the neck medially at the midline and laterally to the anterior border of the sternocleidomastoid muscle. The flap is evaluated for skin laxity and marked accordingly to facilitate primary closure of the defect. The flap markings extend from the hyoid bone to the suprasternal notch. The size of flap varies in the range 3.5–4 cm mediolaterally and 8–10 cm superoinferiorly. A lateral incision is made through the skin and subcutaneous tissue until the deep cervical fascia over the sternocleidomastoid is identified. The deep fascia is incised, and the flap is raised medially to identify the superior thyroid artery and vein. Neck dissection is performed at this point. The superior, medial, and inferior incisions are similarly performed, identifying the ipsilateral strap muscles in the midline and inferiorly at their sternal attachment. Detachment of the sternohyoid, sternothyroid, and omohyoid muscles is done from the hyoid, midline, and sternum, and the flap is elevated. Care is taken to identify and preserve the thyrohyoid muscle, since this helps preserve the superior laryngeal pedicle and internal branch of the superior laryngeal nerve. The flap is raised in the sub-muscular plane islanded on the superior thyroid vessels. The major limitation of the infrahyoid flap is its short reach, since its reach is dependent upon the location of the superior thyroid pedicle.

#### The platysma myocutaneous flap

**Figure 7:**
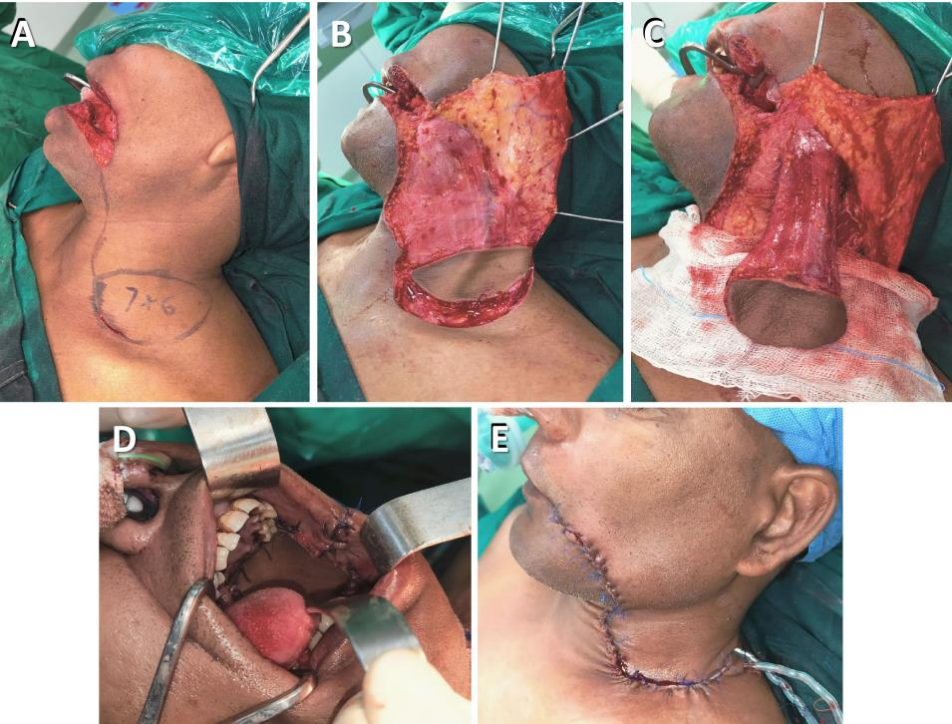
Platysma Flap for Complex Buccal Mucosa Defect Reconstruction. **A:** Marking of flap and neck dissection. **B:** Flap elevation. **C:** Flap islanded and neck dissection completed. **D:** Flap inset into defect. **E:** Closure of defect and neck.

Figure 7 shows the steps involved when using the PMF.

The PMF is marked so that a horizontal incision for neck dissection becomes the lower border of the flap. The lower incision is made through the skin, subcutaneous tissue, and platysma, whereas the upper incision goes through the subcutaneous tissue. The upper neck flap is elevated in the supraplatysmal plane until reaching the lower border of the mandible. The rest of the flap is raised in the subplatysmal plane, incorporating the investing layer of deep cervical fascia on the sternocleidomastoid muscle and external jugular vein. During neck dissection the submental vessels are preserved. The flap is rotated superiorly and lateral to the mandible and positioned in the oral defect. Care should be taken to avoid twisting or compressing the flap, which would compromise the blood supply and lead to flap congestion.

The PMF is based primarily on the submental artery and the external jugular vein.^22^ However, the venous blood supply of the flap is less reliable, and preserving its vascularity while performing a neck dissection is more challenging.

## LIMITATIONS

Our study had a few inherent limitations. Being retrospective in nature, the patient complications were evaluated for only a short-term duration of follow-up. This was a comprehensive review of 104 different local flap cases, their surgical techniques, and perioperative complications (up to 6 months). Ideally, a parallel arm prospective comparative study between local flaps and free flaps could provide better insight into the oral cavity defect reconstructions. This study did not evaluate the long-term functional aspects such as swallowing, trismus, donor site scar assessment, and quality of life evaluation.

## CONCLUSION

Within the limitations of the study, small to medium-sized oral cavity defects can be reconstructed with local flaps with relatively few instances of donor site morbidity. In our experience, local flaps can be successfully used for complex defect reconstructions like anterior two-thirds glossectomy, floor of mouth defects, and buccal mucosa defects with alveolectomy or marginal mandibulectomy. These patients could receive timely surgery despite the limitations of care within high-volume centers with limited free flap reconstruction resources. This study highlighted the versatility of local flaps and their undervalued potential for small to medium-sized reconstructions of oral cavity defects.

The learning curve for utilizing local flaps in complex oral cavity defects is relatively steep. The surgeon must be well-versed in the vascular anatomy of the face and neck. Furthermore, patient selection and assessment of the defect are crucial, followed by meticulous planning. Primary closure of the donor site is preferable; donor site morbidity should be minimal. Node-negative status of the neck is preferable, and oncological safety is foremost in the selection of local flaps. Ultimately, utilization of local flaps must be further evaluated by measuring their functional and aesthetic outcomes.

## Abbreviations

FAMM: facial artery myomucosal
PMF: platysma myocutaneous flap.
